# Optimizing the implementation of a population panel management intervention in safety-net clinics for pediatric hypertension (The OpTIMISe–Pediatric Hypertension Study)

**DOI:** 10.1186/s43058-020-00039-z

**Published:** 2020-06-25

**Authors:** Justin D. Smith, Nivedita Mohanty, Matthew M. Davis, Ashley A. Knapp, Yacob G. Tedla, Allison J. Carroll, Heather E. Price, Juan A. Villamar, Roxane Padilla, Neil Jordan, C. Hendricks Brown, Craig B. Langman

**Affiliations:** 1 Departments of Psychiatry and Behavioral Sciences, Preventive Medicine, Medical Social Sciences, and Pediatrics, Northwestern University Feinberg School of Medicine, Chicago, IL USA; 2 Department of Pediatrics, Northwestern University Feinberg School of Medicine, Chicago, IL USA; 3 Stanley Manne Children’s Research Institute, Ann & Robert H. Lurie Children’s Hospital of Chicago, and Departments of Pediatrics, Medicine, Medical Social Sciences, and Preventive Medicine, Northwestern University Feinberg School of Medicine, Chicago, IL USA; 4 Department of Psychiatry and Behavioral Sciences, Northwestern University Feinberg School of Medicine, Chicago, IL USA; 5 Department of Medicine, Northwestern University Feinberg School of Medicine, Chicago, IL USA; 6 Department of Psychiatry and Behavioral Sciences, Northwestern University Feinberg School of Medicine, Chicago, IL USA; 7 Stanley Manne Children’s Research Institute, Ann & Robert H. Lurie Children’s Hospital of Chicago and Department of Pediatrics, Northwestern University Feinberg School of Medicine, Chicago, IL USA; 8 Department of Psychiatry and Behavioral Sciences, Northwestern University Feinberg School of Medicine, Chicago, IL USA; 9 Departments of Psychiatry and Behavioral Sciences, Preventive Medicine, and Medical Social Sciences, Northwestern University Feinberg School of Medicine, Chicago, IL USA; 10 Ann & Robert H. Lurie Children’s Hospital of Chicago and Department of Pediatrics, Northwestern University Feinberg School of Medicine, Chicago, IL USA

**Keywords:** Children, Adolescents, Blood pressure, Hypertension, Pediatric, Population health, Youth

## Abstract

**Background:**

Though clinical practice guidelines are available, the diagnosis of pediatric hypertension (HTN) is often missed. Management may not follow guidelines due to the measurement challenges in children, complexity of interpreting youth blood pressure standards that are dependent on height, age, and sex, familiarity with diagnostic criteria, and variable comfort with management of pediatric HTN among providers. Evidence suggests that wide adoption and adherence to pediatric HTN guidelines would result in lower cardiovascular disease and kidney damage in adulthood. The proposed project will develop an implementation strategy package to increase adherence to clinical practice guidelines for pediatric HTN within safety-net community health centers (CHCs). The centerpiece of which is a provider-facing population panel management (PPM) tool and point-of-care clinical decision support (CDS). Prior research indicates that multiple discrete implementation strategies (e.g., stakeholder involvement, readiness planning, training, ongoing audit and feedback) are needed to institute practice- and provider-level adoption of such tools.

**Methods:**

Using participatory research methods involving stakeholders from a practice-based research network of CHCs, with input from scientific advisors, the project aims to (1) employ user-centered design methods to tailor an existing CDS tool for use at the point of care and optimize cohort management with a PPM tool to support adherence to the latest pediatric HTN guidelines, and (2) use a stakeholder-driven method for selecting implementation strategies that support tool adoption and increase guideline-adherent physician behaviors. Multilevel process evaluation using surveys and key informant interview data will assess the acceptability, adoption, appropriateness, cost, and feasibility of the PPM tool and its multicomponent implementation strategy package. Usability testing will be conducted with the PPM tool to iteratively refine features and ensure proper functionality.

**Discussion:**

The proposed research has the potential to improve identification, diagnosis, and management of HTN in primary care settings for high-risk youth by assisting healthcare providers in implementing the American Academy of Pediatrics’ 2017 guidelines using an EHR-integrated PPM tool with CDS. Should the strategy package for PPM tool adoption be successful for pediatric HTN, findings will be translatable to other settings and PPM of other chronic cardiovascular conditions affecting overall population health.

Contributions to the literature

- The OpTIMISe–Pediatric Hypertension Study protocol involves a stakeholder-driven approach for the design of health information technology tools to support guideline-adherent care of pediatric hypertension, an underdiagnosed condition with significant health disparities, as well as the development of a multicomponent implementation strategy to support uptake by pediatric providers.
- This protocol is unique in its use of user-centered design and stakeholder-engaged methods in the implementation preparation phase and its focus on both population-based and point-of-care health information technology strategies to support implementation.
- This study is conducted in safety-net community health centers serving socioeconomically disadvantaged children who are at high risk for cardiovascular disease.

## Background

Target organ damage, especially left ventricular hypertrophy associated with heightened risk for cardiovascular events in adults, is detectable in youth with primary hypertension (HTN) [1, 2]. Risk factors for adult HTN and concomitant target organ damage from HTN have been shown to begin in childhood [3–5]. Hypertensive children are more likely to have adult hypertension and metabolic syndrome [5, 6]. Primary prevention is critical given estimates that almost 10% of HTN in adults could be prevented if high blood pressures (BP) in childhood were recognized and treated [7–10]. The need to diagnose and manage HTN is further magnified due to increasing rates of childhood obesity [11–13] that indicate disproportionate effects on those who are disadvantaged economically and socially [14–16]. Guidelines for pediatric HTN were issued by the American Academy of Pediatrics (AAP) in 2017 [17] yet the diagnosis of pediatric HTN is often missed, and management may not follow the guidelines due to the complexity of interpreting youth BP standards, diagnosing HTN, and variable familiarity with managing pediatric HTN among pediatric healthcare providers [18].

A recent study in community health centers in the Chicago area found only 6.1% among 1478 children (age ≥ 3 and < 18 years) who met criteria for abnormal BP—based on BP values from clinical encounters recorded in the electronic health record (EHR)—were correctly diagnosed [19]. Evidence suggests that wide adherence to the HTN guidelines would result in lower risk for target organ damage to the heart and the kidneys and HTN-related cardiovascular disease in adulthood [17]. There is a pressing need for effective implementation strategies to ensure adherence to the pediatric HTN guidelines in primary healthcare systems, particularly those that serve high rates of children and adolescents at greatest risk for HTN due to the health disparities associated with obesity and or low birth weight [20–22].

### Pediatric HTN and co-occurring cardiovascular conditions are a public health concern

In the USA, nearly 1 in 9 children exhibit serious cardiometabolic symptoms, including HTN, type 2 diabetes, insulin resistance, non-alcoholic fatty liver disease, asthma, and obstructive sleep apnea, all of which accelerate the risk for cardiovascular disease and mortality [23]. Additionally, cardiometabolic risk factors in youth tend to cluster [24]. Both the Bogalusa Heart [4] and Fels Longitudinal [5] studies clearly demonstrated that greater numbers of individual elevated BP measurements in childhood confer increased risk of adult HTN. Two recent cross-sectional studies further indicate that target organ damage is also detectable in adolescents with pre-HTN as well as HTN [25, 26].

About 3% of the general population has HTN, while about 25% of youth with obesity (BMI ≥ 95^th^%) have HTN [27]. A study examining childhood HTN and overweight/obesity in school children saw that 2.2% of the sample had HTN, and 37% of HTN cases could be attributed to overweight/obesity [28]. In a meta-analysis examining cardiovascular risk factors, compared with normal weight children, systolic BP was higher by 4.54 mmHg (99% confidence interval 2.44 to 6.64; *n* = 12,169, 8 studies) in overweight children, and by 7.49 mmHg (3.36 to 11.62; *n* = 8074, 15 studies) in children with obesity [29].

Additionally, the economic impact of pediatric HTN is substantial. There is evidence that elevated BP among children is associated with higher care costs: in a cohort of children and adolescents aged 3 to 17 years who received preventative care services in two states, youth with HTN had significantly higher annual health care costs ($1972 average) than those with normal BP ($736) or pre-HTN ($945), after adjusting for body mass index [30].

### Cardiovascular health is an indicator of health disparities

According to the most recent estimates from the 2015–2016 National Health and Nutrition Examination Survey, 18.5% of all 2- to 19-year-old youth are obese and that a disproportionate number are disadvantaged economically and socially [31]. Obesity and its cardiovascular health consequences are disproportionately distributed across the USA; Mexican Americans, American Indians, and African Americans have the highest prevalence rates [32–35]. Low-income youths bear the burden, with risk factors at family and neighborhood levels [36, 37]. Stress and discrimination, both acutely experienced by minority and low-income communities in the USA, have been linked with cardiovascular health [38]. To address this, effective implementation of best practices for preventive care and chronic disease management is needed in safety-net health systems.

### Pediatric HTN is underdiagnosed with incomplete management

Unlike the definition of adult HTN, which is linked to a single BP level, pediatric HTN until age 13 is dependent upon multiple factors, including age, gender, and stature [17]. Knowledge of the threshold values for acceptable BP recordings up until age 13 is therefore dependent on the clinician taking the BP reading and finding the corresponding BP or HTN category for that child. Diagnosis of HTN is further dependent upon taking multiple BP readings, whose interval of repetitive measurements is dependent upon the initial readings themselves. About 48.2% of stage I HTN diagnoses are correct, and only 35.0% of stage II HTN diagnoses are correct [39]. Thus, follow-up appointments must be carefully coordinated to assure accuracy.

The ideal methods of BP recording require having the patient in a quiet room, sitting, and without verbal interactions [17]. The majority of BP readings performed in an office setting initially use an oscillometric device that is often inaccurate compared to a mercury-based sphygmomanometer [17]. These specifications place a high burden on pediatric clinicians and their assistants in typical practices. Management of suspected HTN based on the new guidelines further requires a 24-h ambulatory BP study as well as an echocardiogram, but most general pediatric clinicians do not have such resources easily available to them, requiring referral to specialists. Last, most clinicians caring for children are unfamiliar with pharmacologic management of HTN—again requiring a referral to a sub-specialist. As a result, abundant evidence indicates that pediatric HTN is underdiagnosed.

### The 2017 AAP guidelines for pediatric HTN are challenging to implement

**Fig. 1:**
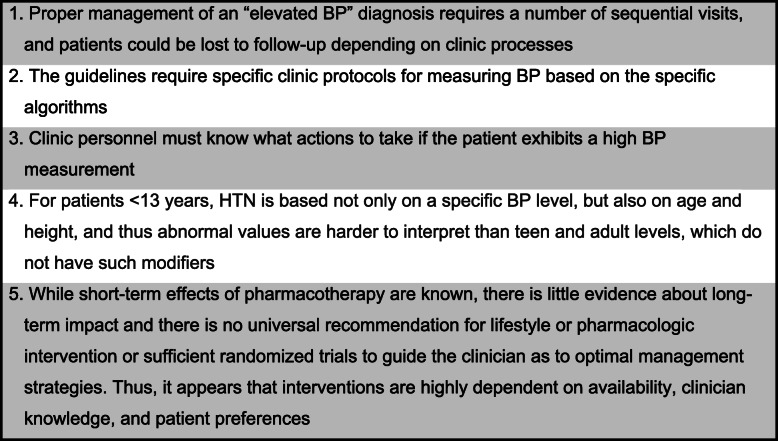
Challenges for implementing current AAP guidelines for youth hypertension

The newest guidelines for clinical care of pediatric HTN [17] (referred to in the guidelines as elevated BP) has 30 Key Action Statements and 20 Tables for clinicians to understand and implement, making it very difficult to satisfy at a single office visit in which a BP reading may be above a reference threshold. As a result, many studies show primary care providers are not adherent, particularly related to screening and identification of abnormal BP [16, 40]. To reiterate, challenges of the latest guidelines include calling HTN “elevated blood pressure”, and the issues presented in Fig. 1.

### Health information technology to increase guideline adoption and adherence

This project focuses on optimizing cohort management with a population panel management (PPM) tool and tailoring an existing clinical decision support (CDS) tool for use at the point of care. PPM tools consist of a primary care practitioner or staff identifying patients who have unmet preventive and chronic care needs using panel-based health information technology tools [41]. At the point of care, CDS can then provide reminders and prompts to assist providers in managing their patient’s conditions, such as cardiovascular disease, obesity, or HTN. Immunizations are prototypic of a successful CDS system [42].

In the scope of single encounter, it is challenging for providers to review all historical data to make an accurate diagnosis. One of the values of a PPM tool is the ability to visualize data for a panel. As such, PPM tools could remind clinicians to schedule visits [17] and help clinicians identify children who merit a BP re/measurement [17]. A real-time alert tool embedded in EHRs, along with provider education, has been shown to increase recognition of elevated BP in children [43], and providers are more likely to correctly abide by the multiple BP readings protocol and order appropriate tests when CDS is available [44]. In a randomized trial, there was a significantly (*p* < .001) higher rate of correct identification of patients with HTN (54.9%) among clinics with CDS compared to clinics with none (21.3%) [45]. Despite these opportunities, implementation of eHealth tools for PPM and CDS are affected by the technology itself, the inner and outer setting characteristics, and individual health professionals’ readiness to change [46]. Addressing these challenges merits rigorous implementation research and forms the basis of this project.

Adoption of and adherence to the guidelines for pediatric HTN would improve all aspects of underrecognized and inconsistently managed condition that continues to rise amidst the childhood obesity epidemic. The complexity and variation in practice of these guidelines implies that their adoption hinges on effective implementation. PPM tools are a promising strategy that has yet to be evaluated in this context. To speed discovery of an effective practice, data- and stakeholder-driven development of new tools and multicomponent implementation strategies is needed.

The overarching aim of the proposed project is to develop and then optimize an effective and feasible implementation strategy package to increase adherence to clinical practice guidelines for pediatric HTN diagnosis and management and to understand contextual barriers and facilitators. We will target children beginning at age 3, when the AAP guidelines indicate onset of universal screening [17]. Prior research indicates that multiple specific implementation strategies are needed to institute practice- and provider-level adoption of PPM, CDS, and similar health information technology tools [47–49]. We will use a low-cost adaptation [50] of the Expert Recommendations for Implementing Change (ERIC) protocol [51, 52], consisting of a panel of stakeholders and key implementation leaders, to identify an initial implementation strategy package comprising feasible, discrete strategies for the adoption of the PPM and CDS tools and the guidelines starting from the four broad strategy types identified by systematic review: stakeholder involvement, readiness planning, training, and ongoing audit and feedback [46].

## Methods

### Study aims

#### Aim 1: Employ user-centered design methods to refine health information technology tools that address multilevel implementation barriers

With a stakeholder panel (e.g., pediatric providers, practice managers), led by the project team and with input from an external scientific advisory board, we will employ a user-centered design (UCD) approach [53, 54] to tailor an existing CDS tool for use at the point of care and optimize cohort management with a PPM tool, specifically to support adherence to the AAP guidelines for pediatric HTN and meet the needs of stakeholders.

#### Aim 2: Develop a multicomponent implementation strategy package

Using the adapted ERIC process, a community stakeholder panel, again with input from our scientific advisory board, will develop the initial implementation strategy package to address known and probable barriers to adoption of the tool and its effectiveness in changing guideline-relevant physician behavior related to identification and diagnosis of HTN. Health economic methods [55] will be used to evaluate the budget impact of the proposed development activities.

### Study design

The proposed project involves three primary entities: Northwestern University Feinberg School of Medicine, Lurie Children’s Hospital of Chicago, and AllianceChicago Community Health Services. Within AllianceChicago are four CHC organizations in the Chicago area that have a long history of involvement in research and practice change initiatives using the AllianceChicago’s shared health information technology infrastructure. These organizations have more than 40 total clinics (range: 9 to 17 clinic locations per organization) and serve predominantly racial/ethnic minority patients (49% Black; 37% Hispanic/Latino; 4% Asian; 8% Non-Hispanic White; 2% Other) with 83% of patients below 100% of the poverty line and 25% uninsured. The demographics of these patients align with the health disparities in HTN per epidemiologic data [27].

The proposed study uses a community-engaged implementation research approach with a user-centered, stakeholder-driven approach when developing the PPM and CDS tools for pediatric HTN and the implementation strategy package. We focus on the impact of specific implementation strategies on provider behaviors as this is consistent with the purpose and targets of the AAP guidelines for pediatric HTN. The link between these provider practices in the guidelines and the clinical benefit to children was determined by the AAP when specifying the guidelines, based on the best available evidence [17]. The implementation *process* is guided by the adapted ERIC protocol [50] and the recommendations of Ross et al. [46]; assessment of *determinants* (barriers and facilitators) is guided by the Consolidated Framework for Implementation Research (CFIR) [56]; and evaluation of *outcomes* is guided by the Proctor et al. [57] taxonomy.

**Fig. 2:**
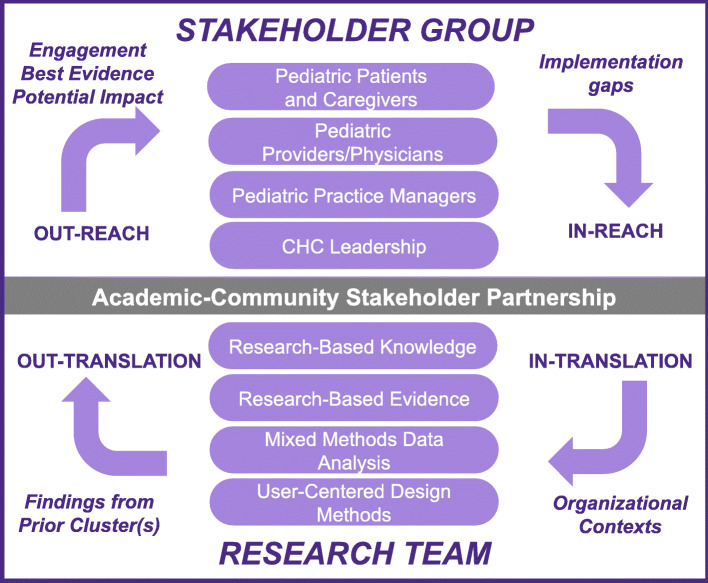
OIIO Stakeholder Engagement Model

Drawing on principles of community-based participatory research [58] and NHLBI-endorsed community-engaged implementation research methods [59], this project follows the Out-reach, In-Reach, In-Translation, and Out-Translation (OIIO) model [60] (Fig. 2), developed by Co-Author Davis [60], to ensure a strong process of engagement with our partner CHC partners and community stakeholders by ensuring a bi-directional and recursive process of engaging community stakeholders in the development and evaluation activities in the proposed project. The OIIO model will guide the process of the stakeholder panel meetings for PPM implementation strategy development by first eliciting members’ perspectives (“in-reach”) and then sharing findings as they emerge (“out-reach”). The initial *out-reach* step begins by connecting the research team with the members of the stakeholder panel to respectfully and actively promote communication and engagement. The research team will elicit information regarding the needs and knowledge gaps from these diverse stakeholder advisors, which in-turn forms the *in-reach*. In particular, the team will lead *in-translation* of stakeholder needs and implementation gaps into scientific research/scientific concepts, which then guides the research team in framing *out-translation*. The research team shares quantitative and qualitative findings with the stakeholder panel via *out-reach*—bringing together academic-stakeholder knowledge as recursive OIIO cycles iterate over the project period.

#### Implementation preparation (study months [M] 1–12)

The current project consists of the development and planning activities prior to testing the implementation strategy and the PPM tool with pediatric patients in a subsequent study.

#### Recruitment and composition of the stakeholder panel

The stakeholder panel, comprising 6 to 10 members from four CHC organizations, will be recruited in M1 in collaboration with the leadership of each participating organization and through presentations and individual contacts by research staff. Members will be primarily practitioners who see pediatric patients, including physicians, advanced practice providers, and nurse practitioners; those with clinical leadership roles (e.g., chief medical officer) will also be eligible.

#### Tailoring the PPM and CDS tools for pediatric HTN and AAP guidelines

This phase will utilize UCD procedures tailoring an existing CDS tool for use at the point of care and optimize cohort management with a PPM tool (developed by Health Catalyst) already in use within AllianceChicago for adults with diabetes, HTN, HIV, and Medicare chronic care management. UCD is an iterative development process involving multiple cycles of evaluation and refinement of prototypes through deep engagement with relevant stakeholders [61]. AllianceChicago is well-versed in UCD procedures for health information technology tools. To facilitate engagement, the stakeholder panel will convene for a 2-h co-design workshop in M2 and M4 to discuss and provide feedback on the tailoring of the PPM and CDS tools’ initial features, functions, and required data inputs. The initial design will be used to start another cycle of co-design workshops. We anticipate that the majority of adaptation changes will happen within the first three iterations [54]. Thus, we plan to conduct at least three iterations of UCD meetings (four are planned; M3-M6) to ensure we capture all the major adaptations for the PPM tool while adhering to the AAP guidelines.

As features and functions are identified by the stakeholder panel, AllianceChicago will begin to build the tools (M3–M7). This is followed by two phases of usability testing, performed following the same procedures. In M7–M8, 10 participants from participating CHCs will evaluate the tools for 30 min in lab-based usability testing. Ten participants are typically required to identify 95% of usability problems [62]. Participants will be videotaped while using a “think aloud” framework as they use the tools to complete a set of basic use tasks [63]. At the conclusion of the testing session, participants will complete a modified version of the System Usability Scale [64] to assess the technology and will be asked to engage in brief user feedback interviews to identify (1) if user goals were met, (2) if any problems or difficulties were encountered, and (3) suggestions for improvements. Following lab-based usability testing of the tools, there will be an integration period with each of the CHC organizations’ EHR systems and functionality testing (M9–M10). Although the tools will be the same across all organizations because of the shared AllianceChicago infrastructure, patient and provider data for PPM will need to be specified for each organization. Once the PPM and CDS tools are installed, the 10 participants will engage in the same usability procedure and assessment once again. All major problems identified during both usability tests will be fixed prior to testing the tool in practice in a subsequent study.

#### Developing and piloting the implementation strategy package for the health information technology tools

The standard practice when AllianceChicago implements a new health information technology feature is to activate the associated EHR tools, provide written instructions and webinars that explain the tool to its members, and email members to alert them that new tools are in place. A large body of research suggests that such strategies alone without commensurate site-level training are unlikely to result in efficient and complete adoption of the PPM and CDS tools [46], and even intensive training is insufficient to achieve high rates of adoption and use of such tools [46]. This study will develop a *package* of implementation strategies (often referred to as a toolkit) to support uptake of the tools and use with fidelity.

**Fig. 3:**
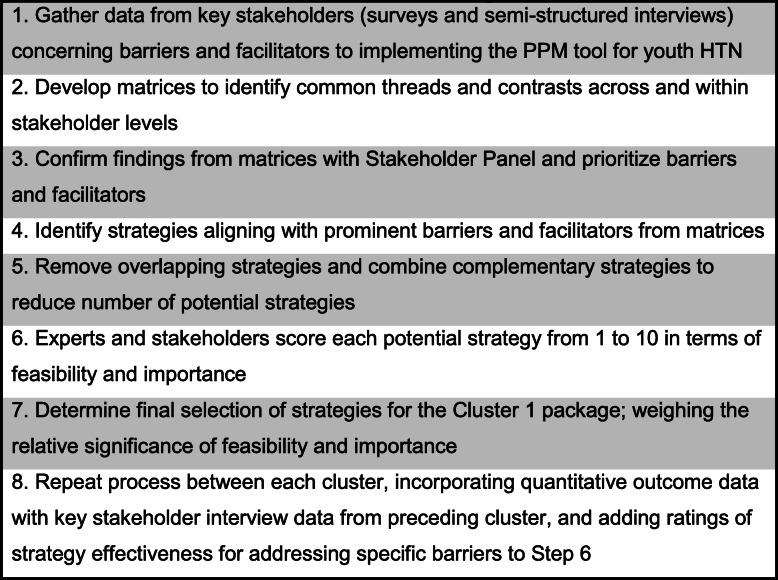
The adapted ERIC procedure

We propose to follow the protocol of Go et al. [50], a pragmatic adaptation of the ERIC protocol [51, 52, 65]. In M5, the stakeholder panel will begin to develop the initial implementation strategy package following the procedures in Fig. 3. We will use a sequential mixed methods approach involving brief surveys (Step 6 in Fig. 3) of the stakeholder panel members followed by key informant interviews. Results will form the basis of a matrix used to identify common threads and contrasts across and within stakeholder levels. This matrix of common barriers and facilitators is then used in panel meetings to identify the specific type and intensity of implementation strategies to address them. The ERIC matrices are used to advance our understanding of the specific barriers and facilitators to adoption, to inform the process of prioritization with stakeholders across organizations, and to identify feasible implementation strategies through voting and an iterative dialog with our stakeholder panel. Rather than a single occurrence, we will repeat the adapted ERIC procedures to capture emergent barriers and tailor the strategies.

**Fig. 4:**
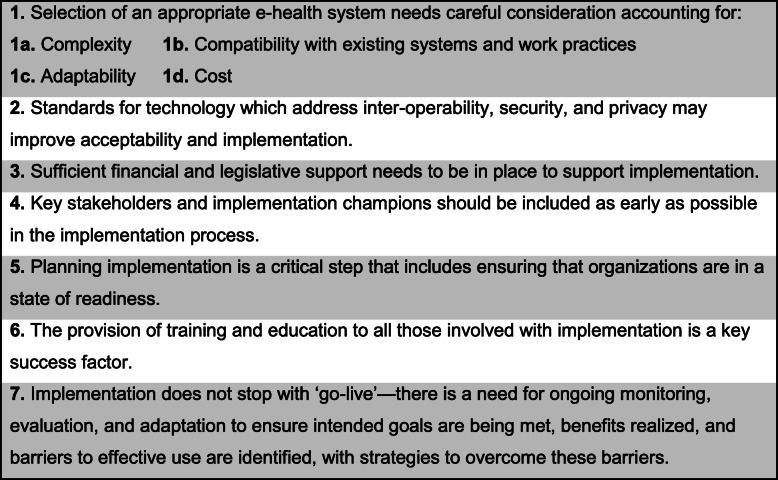
Recommendations for the implementation of eHealth innovations

To begin the modified ERIC process from an evidence-based foundation, we plan to use the results and recommendations from a recent systematic synthesis of 44 published reviews on eHealth implementation [46], focused on EHR, CDS, and PPM tools in primary care (Fig. 4). The strategy selection and incorporation process are described in detail in Additional file 1. Briefly, the development of the PPM tool for pediatric HTN will follow co-design workshops (Aim 1) explicitly focusing on these aspects. Strategies 2 and 3 are already in place and facilitated by AllianceChicago’s shared health information technology infrastructure. Strategies 4–7 will be the focus of the ERIC process for optimization. Concerning Strategy 4, we have designed our study activities to prospectively involve stakeholders and champions in the design of the tool and the initial implementation strategy package. Planning (Strategy 5) is also built into the study design and the process of developing and optimizing the implementation strategy and can also be modified with each successive cluster rollout. For training and education (Strategy 6), numerous discrete implementation strategies exist which offer significant adaptability for the needs of the intervention [65]. Finally, for Strategy 7, we will follow best practices for audit and feedback [66–68]. We will use established guidelines [69–71] to ensure accurate and detailed specification of the strategies used within the package as part of the mixed methods analytic plan.

Beyond Ross et al.’s summary, we anticipate using strategies unique to a PPM tool. The primary functionality of a PPM tool that distinguishes it from CDS and other eHealth interventions is the ability to examine cohorts of the clinic population for “missed opportunities” in guideline adherence. This population-based snapshot, as opposed to a single patient (i.e., point-of-care) perspective, allows clinic staff to examine recent and upcoming patient visits, as well as patients inactive in care, for indicators of need for outreach to re-engage in care for attention to HTN-related concerns. The precise manner in which these unique aspects of the PPM tool will be used in this study are unknown given the dearth of such tools for children.

The adapted ERIC process of barrier assessment, strategy selection, and optimization will be further organized through the use of the Implementation Research Logic Model [72, 73] as a process evaluation tool to depict iterative changes over time, and causal pathway diagrams [74]. Both of these methods specify the associations between identified barriers, specific implementation strategies, the mechanism(s) by which the strategy addresses the barrier, and the desired outcomes.

### Assessment strategy, measures, and data analysis

The proposed study incorporates surveys and stakeholder interviews/meetings with multiple respondents (e.g., providers, key stakeholders). Mixed methods approaches for implementation research [75–77] are central to evaluating the tailoring and optimization of the PPM and CDS tools (Aim 1) and the implementation strategy package (Aim 2).

#### Aim 1: Tailor CDS and optimize PPM tools that addresses multilevel implementation barriers

The co-design workshops (*n* = 2) and stakeholder panel meetings (*n* = 2 meetings), and the “think aloud” portion of usability testing sessions (*n* = 20 total), will be evaluated through a sequential mixed methods approach involving rapid qualitative analysis of video recordings alongside quantitative results of the System Usability Scale [64]. In this project, the CFIR [56] interview guide will be used to semi-structure the stakeholder panel meetings. To ensure that qualitative results can be processed within our timeframe, we use a quick and comprehensive qualitative analysis strategy called Framework-Guided Rapid Analysis [78] in which a structured template based on the CFIR [56] analysis template is generated. To further reduce the necessary time for qualitative data processing, we will (1) identify themes and conduct content analysis directly from audio recordings, which can be done without the need for time-consuming transcription [79], and (2) provide “summary templates” rather than the typically lengthy results from intensive coding. Implementation researchers have used this method successfully when quick turn-around is required [80]. These qualitative analysis methods have been found to be reliable while requiring only about 13% more time than the recording [79], and some have found that a rapid approach is comparable to thematic analysis in terms of identifying key actionable findings [81]. Use of the CFIR interview guide and analysis template ensure that aspects of the domains and subdomains mentioned by the panel are captured, and uniform terminology and the definitions of other implementation researchers are used, which will increase rigor and reproducibility.

All qualitative data processing and analysis will be done by a trained Research Associate and a Research Assistant, overseen by JS. For reliability, 20% of panel meetings and “think aloud” tasks will be double-coded in order to calculate reliability [82]. Disagreements in coding will be resolved via expert consensus among the investigative team led by JS, who has led studies using qualitative and mixed methods [83–85] and rapid qualitative analysis methods and the CFIR tools. The mixed methods analytic approach in this aim will be a “merge the data” approach described by Palinkas and Cooper [75], which involves bringing together quantitative and qualitative data through complementarity [76, 77].

#### Aim 2: Develop an implementation strategy package

Evaluation of the strategy development will be mixed methods involving qualitative data analysis from key informant interviews and the stakeholder panel meetings and the results of quantitative surveys.

#### Qualitative analysis of the optimization process

Procedures for the collection, rapid analysis, and reliability of qualitative data are described in Aim 1. To ensure detailed reporting of the implementation strategy package, we will follow published guidelines [69] and use the Implementation Research Logic Model [72, 73]. This is critical for interpretation and rigor and reproducibility of the study, as well as costing of these activities (see “Cost analysis” section below).

#### Quantitative analysis of the development process

Surveys administered during development activities of this project focus on usability of the tool (see Aim 1) and the feasibility, appropriateness, and acceptability of the tool and its implementation strategy package assessed via brief, validated surveys developed by Weiner et al. [86] after tool and strategy package development.

#### Mixed methods analysis

The mixed methods analytic approach is sequential (“Connect the dots”) [76, 77] for integrating the qualitative data with the quantitative data. This approach will provide a detailed evaluation of the development process and the specific strategies involved in the package.

#### Cost analysis

Economic analyses are central to the implementation evaluation and for providing data of use to potential adopters (e.g., administrators, policy makers). Our cost evaluation within this project will focus on estimating the costs associated with implementing each discrete strategy and the package of strategies developed by the stakeholder panel. This cost analysis will be conducted from the perspective of a provider organization that may wish to adopt the multicomponent strategy developed in this study. An ingredient-based cost analysis procedure, including costs for tool refinement and optimization and integrating it with the EHR, training providers and other stakeholders, time of provider usage, and other personnel time to support implementation, will be employed to estimate costs associated with each discrete strategy. Strategy costs will be aggregated to estimate total package costs. Consistent with the aims of this project, we will estimate total cost of implementation preparation activities (that is, the activities required to get ready for implementation prior to any patient benefit) as has been done in prior implementation studies led by NJ and JS [87, 88]. Using an activity-based costing approach will allow us to value activities both locally (Chicago, Illinois) and from national data sources (e.g., Current Population Survey, US Department of Labor), providing estimates relevant for scale up in new pediatric care settings nationwide.

#### Normalization

Defined as the perception of *potential* standardization of the PPM and CDS tools, normalization will be assessed using the NoMAD instrument [89, 90]. The NoMAD assesses staff perceptions of factors relevant to embedding health information technology that changes typical work practices. Based on Normalization Process Theory [91], the 23-item NoMAD is concerned with: *implementation* (bringing a practice or practices into action), *embedding* (when a practice or practices may be routinely incorporated into everyday work), and *integration* (when a practice or practices are reproduced and sustained in the social matrices of an organization) [89]. The NoMAD is particularly relevant for evaluating practice changes in primary care involving health information technology interventions [92–94]. It will be administered to all involved stakeholders (physicians/providers/practice leaders) in M11.

### Protocol modifications and current status

Based on feedback from our scientific advisory board, we have made three modifications to the protocol. First, to provide contemporary baseline adherence rates to the guidelines prior to implementation, we will perform two secondary data analyses of EHR data from the AllianceChicago network of CHCs to characterize the prevalence of correct diagnosis of pediatric HTN and elevated BP. These analyses will be conducted on two time periods. The first will be 1 year prior to the guideline changes (visits occurring between December 2016 and December 2017), and the second period will be the 2 years after the guidelines changed and minimal EHR capacities were added (e.g., automatic calculation of age-specific percentiles). Second, consistent with the goal of Aim 1 (to refine a PPM tool that addresses multilevel implementation barriers), we will invite pediatric patients at risk for HTN and their caregiver(s) to participate in a brief interview to better understand their needs and perspectives around the assessment, diagnosis, and management of pediatric HTN to inform implementation strategies concerning raising awareness and motivating adherence with guideline-based care, such as returning for a repeat BP measurement after a first elevated BP. Third, also aligned with Aim 1, pediatric primary care providers will be invited to complete an online survey to better understand their perspectives around identifying and managing pediatric HTN. Feedback from the patient-caregiver interviews and provider surveys will help us better design and implement the PPM tool and associated implementation strategies to support guideline-adherent care for HTN.

At submission of this protocol, the project is well underway. Specifically, we have recruited the stakeholder panel and the meeting dates have been scheduled; developed and pilot-tested the provider survey; and convened a meeting of the Scientific Advisory Board. Further, the first set of secondary data analyses of EHR data have been conducted and a manuscript is currently being prepared for submission.

It is worth mentioning that we are currently in the middle of the COVID-19 pandemic in the state of Illinois, which includes a stay-at-home order. AllianceChicago CHCs, like most healthcare systems across the nation, are shifting their focus to managing the pandemic. Accordingly, we have temporarily suspended administration of the provider survey and will hold meetings of the stakeholder panels virtually. Our team will continue to modify the protocol as required to meet the shifting needs of our stakeholders and collaborators in the healthcare system during this pandemic, while also adhering to public health guidelines, guidance from Northwestern University, and recommendations from the Center for Disease Control and Prevention and the Illinois and Chicago Departments of Public Health.

## Discussion

The ultimate goal of the proposed project is to develop a feasible implementation strategy package to increase adherence to clinical practice guidelines for pediatric HTN diagnosis and management. We will accomplish this aim using a PPM tool and CDS at the point of care. This will broaden the reach of HTN diagnosis and management by providing a tool that assists providers and benefits an entire patient population.

The protocol will extend the existing literature in two novel ways. First, we will design and implement a PPM tool to support the adoption of AAP guidelines for the diagnosis and treatment of pediatric HTN. No such sophisticated, EHR-integrated tool currently exists for pediatric HTN, despite evidence that PPM tools are effective at improving diagnosis and management of multiple chronic health conditions (including HTN in adults), and PPM tools are unique and underutilized tools for guideline implementation in pediatrics. Additionally, while the majority of existing PPM tools are for adults, this innovation will focus on children and adolescents. Second, we will leverage an innovative stakeholder-driven process for tool development using UCD methods and development of an implementation strategy package based on best available evidence from the field of implementation science and a low-cost adaptation of the ERIC process. Third, we will conduct a prospective implementation cost evaluation informed by stakeholders to ensure that critical questions informing economic sustainability and incremental cost-benefits are answered. Inadequate cost evaluation is a top reason for failure to implement [95], and an insufficient proportion of implementation research studies include prospective economic evaluation [96]. Cost information is highly informative for potential adopter organizations and can be used to quanitify the monetary investment required to achieve adoption and the related clinical outcomes of interest.

We anticipate two specific challenges while conducting this study. First, the HTN guidelines currently require repeated measurement of pediatric patients 'BP. As discussed, this guideline receives low adherence among pediatric healthcare professionals who are managing large workloads and competing priorities. Further, it places a large burden on families to attend multiple medical appointments. The activities in Aim 2 will actively address this challenge through understanding the barriers specific to providers and families around this aspect of the AAP pediatric HTN guidelines. Second, accurate assessment of BP in pediatric patients is critical. This speaks to the need of accessible training for pediatric healthcare providers and availability of correct BP assessment tools. Incorporating training in simply measuring BP accurately for younger children (ages 2 to 5 years) and helping pediatric clinics obtain arm cuffs that are properly sized for youth with obesity might become necessary.

Identifying youth with HTN is the gateway to getting them into effective treatments that can prevent the deleterious progression of cardiovascular disease risk. Should the implementation strategy package for PPM tool adoption and guideline adherence be successful for pediatric HTN, findings will be translatable to other settings and for PPM of other chronic heart, lung, blood, and sleep conditions among children and adults, thereby decreasing the health care burden of target organ damage in the cardiovascular system, and other complications, in the adult years and improving overall population health.

## Supplementary information

**Additional file 1.**

**Additional file 2.** Notice of Award. Notice of award from NHLBI – Revision # 1 – Removal of Human Subjects Restrictions.

**Additional file 3.** IRB Approval. Notice of IRB approval from Northwestern University.

**Additional file 4.** Informed Consent. Informed consent documents for study components involving human subjects.

## Data Availability

Data and materials are available upon request to the corresponding author.

## Abbreviations

AAP: American Academy of Pediatrics
BP: Blood pressure
CDS: Clinical decision support
CHC: Community health center
ERIC: Expert Recommendations for Implementing Change
HTN: Hypertension
OIIO: Out-reach, In-Reach, In-Translation and Out-Translation model
PPM: Population panel management tool
UCD: User-centered design
