# Supplementary material for: Optimizing the implementation of a population panel management intervention in safety-net clinics for pediatric hypertension (The OpTIMISe–Pediatric Hypertension Study)
Source: Implement Sci Commun. 2020 Jun 25;1:57. doi: 10.1186/s43058-020-00039-z (PMC7386167; doi:10.1186/s43058-020-00039-z)
Supplement: Supplementary file 1 — Additional file 1. [file 43058_2020_39_MOESM1_ESM.docx]

**Additional File 1**

**Additional Information on the Selection of Implementation Strategies**

Ross et al. [1] found support for 7 broad strategies. They provided a number of recommendations for strategy use based on their synthesis. Figure 4 is a recreation of a table in their article summarizing these recommendations. In contrast, the most recent compilation of implementation strategies for health interventions identified 73 discrete strategies from 9 broad categories [2, 3]. The recommendations of Ross et al. to focus on fewer options would be expected given the nature of eHealth innovations and the contexts in which they are typically implemented. Building off existing recommendations and focusing on 4 primary strategies (see Aim 2) further increases the feasibility of the adapted ERIC process. We expect that barriers may emerge from this process that cannot be addressed by the 4 strategies. Thus, the selection of strategies will not be confined to, but rather focused on, those identified as effective in prior research.

We have carefully designed the proposed study to incorporate each of the evidence-based strategies from Ross et al. Specifically, Strategy 1 (in Figure 4) involves the PPM tool and its usability within the AllianceChicago EHR system. The development of the PPM tool for pediatric HTN will follow co-design workshops (Aim 1) explicitly focusing on these aspects. Strategies 2 and 3 are already in place and facilitated by AllianceChicago’s shared health information technology infrastructure. Strategies 4–7 will be the focus of the ERIC process for optimization. Ross et al.’s recommendations support this as they point to the overarching *type* of implementation strategy for which there are multiple options, such as the specific type, amount, timing, and stakeholders involved in each. For example, the literature indicates that there are at least 12 discrete implementation strategies for Training and Education [3] (Strategy 6) alone, each of which has its own variability on these dimensions. There are also multiple options for ongoing monitoring, evaluation, and adaptation (Strategy 7) of the system after going live. One of the more commonly used strategies is a variant of audit and feedback.

A summary report by the RAND Corporation found that audit and feedback strategies were related to improvements in implementation quality, fidelity, and patient outcomes [4]. But results are not unequivocal, and there are best practice recommendations for audit and feedback, specifically: timely and regular feedback, inclusion of basic implementation outcomes (e.g., reach), availability of data for all stakeholders within an agency, and self-reflection within an agency [4-6]. Precisely *how* these best practices are achieved is central to the optimization process of the proposed project.

Concerning Strategy 4, we have designed our study activities to prospectively involve stakeholders and champions in the design of the tool and the initial implementation strategy package. Additionally, the use of implementation champions within each clinic is a strategy that can be varied during optimization. Champions are another common strategy for health information technology implementation, but their effectiveness varies by their personal and professional characteristics and the specific ways in which they are involved in championing the implementation [7, 8]. Planning (Strategy 5) is also built into the study design and the process of developing and optimizing the implementation strategy as described below and can also be modified with each successive cluster rollout as indicated. Thus, Strategies 4-7 can be manipulated in various ways to address barriers and improve implementation over time. Of note, the repeated use of the adapted ERIC protocol to optimize implementation itself aligns with Strategy 7 in that it is a method for providing feedback to stakeholders to change course as indicated. We will use established guidelines [9-11] to ensure accurate and detailed specification of the strategies used within the package as part of the mixed methods analytic plan.

**References**

1. Ross J, Stevenson F, Lau R, Murray E: **Factors that influence the implementation of e-health: a systematic review of systematic reviews (an update).** *Implementation Science* 2016, **11:**146.

2. Powell BJ, Waltz TJ, Chinman MJ, Damschroder LJ, Smith JL, Matthieu MM, Proctor EK, Kirchner JE: **A refined compilation of implementation strategies: results from the Expert Recommendations for Implementing Change (ERIC) project.** *Implement Sci* 2015, **10**.

3. Waltz TJ, Powell BJ, Matthieu MM, Damschroder LJ, Chinman MJ, Smith JL, Proctor EK, Kirchner JE: **Use of concept mapping to characterize relationships among implementation strategies and assess their feasibility and importance: results from the Expert Recommendations for Implementing Change (ERIC) study.** *Implementation Science* 2015, **10:**109.

4. Mattox T, Kilburn MR: **Supporting effective implementation of evidence-based practices: A resource guide for child-serving organizations.** Santa Monica, CA: RAND Corporation; 2016.

5. Ivers N, Jamtvedt G, Flottorp S, Young JM, Odgaard‐Jensen J, French SD, O'Brien MA, Johansen M, Grimshaw J, Oxman AD: **Audit and feedback: effects on professional practice and healthcare outcomes.** *Cochrane Database of Systematic Reviews* 2012.

6. Colquhoun HL, Carroll K, Eva KW, Grimshaw JM, Ivers N, Michie S, Sales A, Brehaut JC: **Advancing the literature on designing audit and feedback interventions: identifying theory-informed hypotheses.** *Implementation Science* 2017, **12:**117.

7. Shea CM, Belden CM: **What is the extent of research on the characteristics, behaviors, and impacts of health information technology champions? A scoping review.** *BMC Medical Informatics and Decision Making* 2016, **16:**2.

8. Miech EJ, Rattray NA, Flanagan ME, Damschroder L, Schmid AA, Damush TM: **Inside help: An integrative review of champions in healthcare-related implementation.** *SAGE Open Medicine* 2018, **6:**2050312118773261.

9. Proctor EK, Powell BJ, McMillen JC: **Implementation strategies: recommendations for specifying and reporting.** *Implement Sci* 2013, **8**.

10. Campbell M, Katikireddi SV, Hoffmann T, Armstrong R, Waters E, Craig P: **TIDieR-PHP: a reporting guideline for population health and policy interventions.** *BMJ* 2018, **361**.

11. Hoffmann TC, Glasziou PP, Boutron I, Milne R, Perera R, Moher D, Altman DG, Barbour V, Macdonald H, Johnston M, et al: **Better reporting of interventions: template for intervention description and replication (TIDieR) checklist and guide.** *BMJ : British Medical Journal* 2014, **348**.
