# Supplementary material for: Optimizing the implementation of a population panel management intervention in safety-net clinics for pediatric hypertension (The OpTIMISe–Pediatric Hypertension Study)
Source: Implement Sci Commun. 2020 Jun 25;1:57. doi: 10.1186/s43058-020-00039-z (PMC7386167; doi:10.1186/s43058-020-00039-z)
Supplement: Supplementary file 2 — Additional file 2. Notice of Award. Notice of award from NHLBI – Revision # 1 – Removal of Human Subjects Restrictions. [file 43058_2020_39_MOESM2_ESM.pdf]

RESEARCH
Department of Health and Human Services
National Institutes of Health

Federal Award Date: 03/27/2020

NATIONAL HEART, LUNG, AND BLOOD INSTITUTE

Grant Number: 1R56HL148192-01 REVISED
FAIN: R56HL148192

Principal Investigator(s):
JUSTIN D SMITH, PHD

Project Title: Optimizing the Implementation of a Population Panel Management Intervention in Safety-Net Clinics for Childhood Hypertension

Zakrzewski, Andrea
Associate Director
1801 Maple Ave.
2nd Floor, Suite 2410
Evanston, IL 602013149

Award e-mailed to: OSR-Awards@northwestern.edu

Period Of Performance:
Budget Period: 09/20/2019 – 08/31/2020
Project Period: 09/20/2019 – 08/31/2020

Dear Business Official:

The National Institutes of Health hereby revises this award (see “Award Calculation” in Section I and “Terms and Conditions” in Section III) to NORTHWESTERN UNIVERSITY in support of the above referenced project. This award is pursuant to the authority of 42 USC 241 42 CFR 52 and is subject to the requirements of this statute and regulation and of other referenced, incorporated or attached terms and conditions.

Acceptance of this award including the “Terms and Conditions” is acknowledged by the grantee when funds are drawn down or otherwise obtained from the grant payment system.

Each publication, press release, or other document about research supported by an NIH award must include an acknowledgment of NIH award support and a disclaimer such as “Research reported in this publication was supported by the National Heart, Lung, And Blood Institute of the National Institutes of Health under Award Number R56HL148192. The content is solely the responsibility of the authors and does not necessarily represent the official views of the National Institutes of Health.” Prior to issuing a press release concerning the outcome of this research, please notify the NIH awarding IC in advance to allow for coordination.

Award recipients must promote objectivity in research by establishing standards that provide a reasonable expectation that the design, conduct and reporting of research funded under NIH awards will be free from bias resulting from an Investigator’s Financial Conflict of Interest (FCOI), in accordance with the 2011 revised regulation at 42 CFR Part 50 Subpart F. The Institution shall submit all FCOI reports to the NIH through the eRA Commons FCOI Module. The regulation does not apply to Phase I Small Business Innovative Research (SBIR) and Small Business Technology Transfer (STTR) awards. Consult the NIH website http://grants.nih.gov/grants/policy/coi/ for a link to the regulation and additional important information.

If you have any questions about this award, please contact the individual(s) referenced in Section IV.

Sincerely yours,

Tyrone A Smith
Grants Management Officer
NATIONAL HEART, LUNG, AND BLOOD INSTITUTE

Additional information follows

NIH NGA R | Version: 56 - 12/26/2018 2:22:00 PM| Generated on: 3/27/2020 12:00:33 AM

SECTION I – AWARD DATA – 1R56HL148192-01 REVISED

Award Calculation (U.S. Dollars)

**Salaries and Wages**	$211,591
**Fringe Benefits**	$55,438
**Personnel Costs (Subtotal)**	$267,029
**Consultant Services**	$1,800
**Travel**	$1,750
**Other**	$3,600
**Subawards/Consortium/Contractual Costs**	$241,896
**Participant Other**	$31,022

**Federal Direct Costs**	$547,097
**Federal F&A Costs**	$191,393
**Approved Budget**	$738,490
**Total Amount of Federal Funds Obligated (Federal Share)**	$738,490
**TOTAL FEDERAL AWARD AMOUNT**	$738,490

AMOUNT OF THIS ACTION (FEDERAL SHARE) $0

SUMMARY TOTALS FOR ALL YEARS		
**YR**	**THIS AWARD**	**CUMULATIVE TOTALS**
1	$738,490	$738,490

Fiscal Information:

CFDA Name: Cardiovascular Diseases Research
CFDA Number: 93.837
EIN: 1362167817A1
Document Number: RHL148192A
PMS Account Type: P (Subaccount)
Fiscal Year: 2019

IC	CAN	2019
HL	8475146	$738,490

NIH Administrative Data:
PCC: HHCEI N / **OC**: 41021 / **Released**: SMITHT 03/25/2020
Award Processed: 03/27/2020 12:00:33 AM

SECTION II – PAYMENT/HOTLINE INFORMATION – 1R56HL148192-01 REVISED

For payment and HHS Office of Inspector General Hotline information, see the NIH Home Page at http://grants.nih.gov/grants/policy/awardconditions.htm

SECTION III – TERMS AND CONDITIONS – 1R56HL148192-01 REVISED

This award is based on the application submitted to, and as approved by, NIH on the above-titled project and is subject to the terms and conditions incorporated either directly or by reference in the following:

a. The grant program legislation and program regulation cited in this Notice of Award.
b. Conditions on activities and expenditure of funds in other statutory requirements, such as those included in appropriations acts.
c. 45 CFR Part 75.
d. National Policy Requirements and all other requirements described in the NIH Grants Policy Statement, including addenda in effect as of the beginning date of the budget period.
e. Federal Award Performance Goals: As required by the periodic report in the RPPR or in the final progress report when applicable.

f. This award notice, INCLUDING THE TERMS AND CONDITIONS CITED BELOW.

(See NIH Home Page at http://grants.nih.gov/grants/policy/awardconditions.htm for certain references cited above.)

Research and Development (R&D): All awards issued by the National Institutes of Health (NIH) meet the definition of “Research and Development” at 45 CFR Part§ 75.2**.** As such, auditees should identify NIH awards as part of the R&D cluster on the Schedule of Expenditures of Federal Awards (SEFA). The auditor should test NIH awards for compliance as instructed in Part V, Clusters of Programs. NIH recognizes that some awards may have another classification for purposes of indirect costs. The auditor is not required to report the disconnect (i.e., the award is classified as R&D for Federal Audit Requirement purposes but non-research for indirect cost rate purposes), unless the auditee is charging indirect costs at a rate other than the rate(s) specified in the award document(s).

This institution is a signatory to the Federal Demonstration Partnership (FDP) Phase VI Agreement which requires active institutional participation in new or ongoing FDP demonstrations and pilots.

Carry over of an unobligated balance into the next budget period requires Grants Management Officer prior approval.

This grant is excluded from Streamlined Noncompeting Award Procedures (SNAP).

This award is subject to the requirements of 2 CFR Part 25 for institutions to receive a Dun & Bradstreet Universal Numbering System (DUNS) number and maintain an active registration in the System for Award Management (SAM). Should a consortium/subaward be issued under this award, a DUNS requirement must be included. See http://grants.nih.gov/grants/policy/awardconditions.htm for the full NIH award term implementing this requirement and other additional information.

This award has been assigned the Federal Award Identification Number (FAIN) R56HL148192. Recipients must document the assigned FAIN on each consortium/subaward issued under this award.

Based on the project period start date of this project, this award is likely subject to the Transparency Act subaward and executive compensation reporting requirement of 2 CFR Part 170. There are conditions that may exclude this award; see http://grants.nih.gov/grants/policy/awardconditions.htm for additional award applicability information.

In accordance with P.L. 110-161, compliance with the NIH Public Access Policy is now mandatory. For more information, see NOT-OD-08-033 and the Public Access website: http://publicaccess.nih.gov/.

This award represents the final year of the competitive segment for this grant. See the NIH Grants Policy Statement Section 8.6 Closeout for complete closeout requirements at: http://grants.nih.gov/grants/policy/policy.htm#gps.

A final expenditure Federal Financial Report (FFR) (SF 425) must be submitted through the eRA Commons (Commons) within 120 days of the period of performance end date; see the NIH Grants Policy Statement Section 8.6.1 Financial Reports, http://grants.nih.gov/grants/policy/policy.htm#gps, for additional information on this submission requirement. The final FFR must indicate the exact balance of unobligated funds and may not reflect any unliquidated obligations. There must be no discrepancies between the final FFR expenditure data and the Payment Management System's (PMS) quarterly cash transaction data. A final quarterly federal cash transaction report is not required for awards in PMS B subaccounts (i.e., awards to foreign entities and to Federal agencies). NIH will close the awards using the last recorded cash drawdown level in PMS for awards that do not require a final FFR on expenditures or quarterly federal cash transaction reporting. It is important to note that for financial closeout, if

a grantee fails to submit a required final expenditure FFR, NIH will close the grant using the last recorded cash drawdown level. If the grantee submits a final expenditure FFR but does not reconcile any discrepancies between expenditures reported on the final expenditure FFR and the last cash report to PMS, NIH will close the award at the lower amount. This could be considered a debt or result in disallowed costs.

A Final Invention Statement and Certification form (HHS 568), (not applicable to training, construction, conference or cancer education grants) must be submitted within 120 days of the expiration date. The HHS 568 form may be downloaded at: http://grants.nih.gov/grants/forms.htm. This paragraph does not apply to Training grants, Fellowships, and certain other programs—i.e., activity codes C06, D42, D43, D71, DP7, G07, G08, G11, K12, K16, K30, P09, P40, P41, P51, R13, R25, R28, R30, R90, RL5, RL9, S10, S14, S15, U13, U14, U41, U42, U45, UC6, UC7, UR2, X01, X02.

Unless an application for competitive renewal is submitted, a Final Research Performance Progress Report (Final RPPR) must also be submitted within 120 days of the period of performance end date. If a competitive renewal application is submitted prior to that date, then an Interim RPPR must be submitted by that date as well. Instructions for preparing an Interim or Final RPPR are at: https://grants.nih.gov/grants/rppr/rppr_instruction_guide.pdf. Any other specific requirements set forth in the terms and conditions of the award must also be addressed in the Interim or Final RPPR. *Note that data reported within Section I of the Interim and Final RPPR forms will be made public and should be written for a lay person audience*.

NIH strongly encourages electronic submission of the final invention statement through the Closeout feature in the Commons, but will accept an email or hard copy submission as indicated below.

Email: The final invention statement may be e-mailed as PDF attachments to: NIHCloseoutCenter@mail.nih.gov.

Hard copy: Paper submissions of the final invention statement may be faxed to the NIH Division of Central Grants Processing, Grants Closeout Center, at 301-480-2304, or mailed to:

National Institutes of Health
Office of Extramural Research
Division of Central Grants Processing
Grants Closeout Center
6705 Rockledge Drive
Suite 5016, MSC 7986
Bethesda, MD 20892-7986 (for regular or U.S. Postal Service Express mail)
Bethesda, MD 20817 (for other courier/express deliveries only)

NOTE: If this is the final year of a competitive segment due to the transfer of the grant to another institution, then a Final RPPR is not required. However, a final expenditure FFR is required and should be submitted electronically as noted above. If not already submitted, the Final Invention Statement is required and should be sent directly to the assigned Grants Management Specialist.

In accordance with the regulatory requirements provided at 45 CFR 75.113 and Appendix XII to 45 CFR Part 75, recipients that have currently active Federal grants, cooperative agreements, and procurement contracts with cumulative total value greater than $10,000,000 must report and maintain information in the System for Award Management (SAM) about civil, criminal, and administrative proceedings in connection with the award or performance of a Federal award that reached final disposition within the most recent five-year period. The recipient must also make semiannual disclosures regarding such proceedings. Proceedings information will be made publicly available in the designated integrity and performance system (currently the Federal Awardee Performance and Integrity Information System (FAPIIS)). Full reporting requirements and procedures are found in Appendix XII to 45 CFR Part 75. This term does not apply to NIH fellowships.

Treatment of Program Income:

Additional Costs

SECTION IV – HL Special Terms and Conditions – 1R56HL148192-01 REVISED

Clinical Trial Indicator: No
This award does not support any NIH-defined Clinical Trials. See the NIH Grants Policy Statement Section 1.2 for NIH definition of Clinical Trial.

REVISION # 1 - REMOVAL OF HUMAN SUBJECTS RESTRICTIONS
This award has been revised to rescind the human subjects restriction placed on the Notice of Award issued 09/17/2019.

NHLBI FUNDING GUIDELINES
This award is being issued in accordance with the NHLBI FY 2019 Operating Guidelines which can be found at: https://www.nhlbi.nih.gov/node-general/fy-2019-funding-and-operating-guidelines

R56 BRIDGE AWARD
This project is awarded for a period of one year, in accordance with the NHLBI Funding Guidelines and the NIH Guide Notice HL-13-188, http://grants.nih.gov/grants/guide/notice-files/NOT-HL-13-188.html.

BUDGET PERIOD
This award is issued for less than a 12-month budget period in order to distribute future noncompeting awards more evenly throughout the fiscal year. Any subsequent noncompeting budget period(s) will begin on 9/1 and will be 12 months in duration. The project period end date has been adjusted accordingly.

STAFF CONTACTS

The Grants Management Specialist is responsible for the negotiation, award and administration of this project and for interpretation of Grants Administration policies and provisions. The Program Official is responsible for the scientific, programmatic and technical aspects of this project. These individuals work together in overall project administration. Prior approval requests (signed by an Authorized Organizational Representative) should be submitted in writing to the Grants Management Specialist. Requests may be made via e-mail.

Grants Management Specialist: Nina Hall
Email: hallnn@mail.nih.gov **Phone**: 301-435-0710 **Fax**: 301-480-3667

Program Official: Catherine Stoney
Email: stoneyc@mail.nih.gov **Phone**: 301-435-6670 **Fax**: 301-480-1773

SPREADSHEET SUMMARY
GRANT NUMBER: 1R56HL148192-01 REVISED

INSTITUTION: NORTHWESTERN UNIVERSITY

Budget	Year 1
Salaries and Wages	$211,591
Fringe Benefits	$55,438
Personnel Costs (Subtotal)	$267,029
Consultant Services	$1,800
Travel	$1,750
Other	$3,600
Subawards/Consortium/Contractual Costs	$241,896
Participant Other	$31,022
TOTAL FEDERAL DC	$547,097
TOTAL FEDERAL F&A	$191,393
TOTAL COST	$738,490

NIH NGA R | Version: 56 - 12/26/2018 2:22:00 PM| Generated on: 3/27/2020 12:00:33 AM

Facilities and Administrative Costs	Year 1
F&A Cost Rate 1	58%
F&A Cost Base 1	$329,988
F&A Costs 1	$191,393
