# Supplementary material for: Optimizing the implementation of a population panel management intervention in safety-net clinics for pediatric hypertension (The OpTIMISe–Pediatric Hypertension Study)
Source: Implement Sci Commun. 2020 Jun 25;1:57. doi: 10.1186/s43058-020-00039-z (PMC7386167; doi:10.1186/s43058-020-00039-z)
Supplement: Supplementary file 3 — Additional file 3. IRB Approval. Notice of IRB approval from Northwestern University. [file 43058_2020_39_MOESM3_ESM.pdf]

Northwestern University
Institutional Review Board
Biomedical IRB
750 N. Lake Shore Dr., 7th Fl.
Chicago, Illinois 60611

irb@northwestern.edu
Office 312. 503. 9338

Social & Behavorial Sciences IRB
600 Foster St., 2nd Floor
Evanston, Illinois 60208

sbsirb@northwestern.edu
Office 847. 467. 1723

APPROVAL OF NEW STUDY

DATE: March 23, 2020

TO: Dr. Justin Smith
FROM: Office of the IRB

DETERMINATION DATE: 3/23/2020
APPROVAL DATE: 3/23/2020

The Northwestern University IRB reviewed and approved the submission described below:

Type of Submission:	Initial Study
Review Level:	Expedited
Expedited Category:	- (5) Data, documents, records, or specimens<br>- (6) Voice, video, digital, or image recordings<br>- (7) Behavioral research/social science methods
Title of Study:	Implementation Preparation of a Population Panel Management Intervention in Safety-Net Clinics for Childhood Hypertension
Principal Investigator:	Justin Smith
IRB ID:	STU00210809
Funding Source:	Name: National Heart, Lung, and Blood Institute
IND, IDE, or HDE:	None
Documents Reviewed:	• SUS_scale.pdf, Category: Questionnaire/Survey;<br>• Optimize_Parent-Permission-with-Child-Assent-CONSENT_3.22.20.docx, Category: Consent Form;<br>• Optimize Flyers Parent Youth_1.27.20.pdf, Category: Recruitment Materials;<br>• Optimize_Parent-Permission-CONSENT_3.22.20.docx, Category: Consent Form;<br>• IRB_Smith NHLBIR01_protocol_3.22.20.docx, Category: IRB Protocol;<br>• Stakeholder Panel Recruitment Letter_2.17.20.docx, Category: Recruitment Materials;<br>• Usability Testing Recruitment Letter_2.17.20.docx, Category: Recruitment Materials;<br>• BP SEW survey_1.27.20 FINAL.docx, Category: Questionnaire/Survey;<br>• stakeholder panel consent_Smith_3.6.20.docx, Category: Consent Form;<br>• phy survey online-consent_Smith English_3.6.20.docx, Category:

	Consent Form;<br>• Optimize usability session consent_3.6.20.docx, Category: Consent Form;<br>• Optmize_Child Assent_3.23.20.docx, Category: Consent Form;<br>• phy survey online Recruitment Letter_2.17.20.docx, Category: Recruitment Materials;<br>• OpTIMISe Dyad Interview Questions_2.18.20.docx, Category: Interview;
Special Determination(s):	Children;

In conducting this study, you are required to follow the requirements listed in the Northwestern University (NU) Investigator Manual (HRP-103), which can be found by navigating to the policy section of the IRB website. Additionally, as Principal Investigator (PI) of this research study, you are expected to adhere to the investigator responsibilities outlined in the "What are my obligations as Investigator in order to conduct Human Research" section of the Investigator Manual (HRP-103).

If your study is a clinical trial, there are additional requirements including trial registration and results reporting on ClinicalTrials.gov. Federally-funded clinical trials are also required to post one IRB approved consent form, used during enrollment, on a publicly available federal website such as ClinicalTrials.gov. Please visit the clinical trials page on the IRB website for more information. If you would like an account created or need other assistance with ClinicalTrials.gov, please email clinicaltrials.gov@northwestern.edu.

An annual continuing review is not required for this project. The study team must still submit: modifications for project changes; RNIs (reportable new information); and a Continuing Review to close the project when it ends (for guidance on when a project can be closed, see GUIDANCE on Study Closure – HRP-1901.

NU IRB approval does not constitute or guarantee institutional approval and/or support. Investigators and study team members must comply with all applicable federal, state, and local laws, as well as NU Policies and Procedures, which may include obtaining approval for your research activities from other individuals or entities.

For IRB-related questions, please consult the NU IRB website at http://irb.northwestern.edu. For general research questions, please consult the NU Office for Research website at www.research.northwestern.edu.

Additionally, please note that the analyst who you worked with during the initial review and approval of your study is not the analyst that is responsible for the review of any subsequent modifications, continuing reviews, or RNIs. As such, please direct any further questions about modifications, continuing reviews, or RNIs to the analyst assigned to the subsequent submission.
