# Supplementary material for: Optimizing the implementation of a population panel management intervention in safety-net clinics for pediatric hypertension (The OpTIMISe–Pediatric Hypertension Study)
Source: Implement Sci Commun. 2020 Jun 25;1:57. doi: 10.1186/s43058-020-00039-z (PMC7386167; doi:10.1186/s43058-020-00039-z)
Supplement: Supplementary file 4 — Additional file 4. Informed Consent. Informed consent documents for study components involving human subjects. [file 43058_2020_39_MOESM4_ESM.pdf]

Title of Research Study: Implementation Preparation of a Population Panel Management Intervention in Safety-Net Clinics for Childhood Hypertension

Principal Investigator: Justin D. Smith, PhD, Associate Professor in the Department of Psychiatry and Behavioral Sciences

Supported By: This research is supported by Northwestern University and the National Heart, Lung, and Blood Institute.

Key Information about this research study:

The following is a short summary of this study to help you decide whether to be a part of this study. Information that is more detailed is explained later on in this form.

- The purpose of this study is to adapt Health Information Technology tools to help providers diagnose and manage abnormal blood pressure of youth ages 3 to 17 years and to inform a strategy for effective implementation of the tools.
- You will be asked to be in a Expert Stateholder Panel with activities over the course of 8 months. The specific activities over the course of these 8 months are co-design workshops, user-centered design and implementation development meetings, usability testing, and in-person meetings.
- There is a potential risk of discomfort, as some of the questions are about personal professional practices and views.
- There are no direct benefits for participation. One potential benefit is that the knowledge gained from this study may contribute to a better understanding of child and adolescent chronic health conditions and may help inform the design of technology tools to better support practicing primary care providers.

Why am I being asked to take part in this research study?

We are asking you to take part in this research study because you are an expert in the field of child hypertension.

How many people will be in this study?

We expect about 8 people will be in this research study.

What should I know about participating in a research study?

- Whether or not you take part is up to you.
- You can choose not to take part.
- You can agree to take part and later change your mind.
- Your decision will not be held against you.
- You do not have to answer any question you do not want to answer.

What happens if I say, "Yes, I want to be in this research"?

Your participation will entail involvement in an Expert Stakeholder Panel with activities over the course 8 months. The panel will convene for:

(1) 2 Co-Design workshop meetings (no more than 3 hours each) in February and March 2020;

(2) 3 user-centered design and implementation strategy development meetings (no more than 3 hours each) in April, May, and June 2020;
(3) a 30-minute usability testing session in your clinic where you will use the tool in a test environment (not with patients) and provide oral feedback and complete a brief 10-item survey about your experience (June and August 2020) (total time is less than 50 minutes).
(4) a 1-hour meeting closing out the panel in late summer or early fall.

All meetings require in-person attendance and will be held at a location close to downtown Chicago. We will ask to video record all meetings as a requirement of your participation so that the study team may later transcribe the meetings to help aid in the design of the Health Information Technology tool.

Is there any way being in this study could be bad for me?

Your participation does not involve any significant risks. There is a potential risk of discomfort, as some of the questions are about personal professional practices and views. You can skip any activities of the Expert Stakeholder Panel you do not wish to participate in, or withdrawal from the study at any point.

What happens if I do not want to be in this research, or I change my mind later?

Participation in research is voluntary. You can decide to participate or not to participate. If you do not want to be in this study or withdraw from the study at any point, your decision will not affect your relationship with Northwestern University. You can leave the research at any time and it will not be held against you. If you decide to withdraw from this study, the researchers will ask you if information already collected from you can be used.

How will the researchers protect my information?

If you participate, all of your responses will be kept confidential. Panel members will be asked to maintain the confidentiality of fellow panel members outside of official meetings. Although we ask everyone in the group to respect the privacy and confidentiality of participants, and to keep the discussion in the group confidential, we cannot guarantee this. Please keep this in mind when choosing what to share in the group setting. All identifying information will be removed from the data collected, which will include qualitative data and survey responses. A de-identified data file will be prepared from the survey data of all respondents and used for analysis and may be shared with the study sponsor. Audio and video recordings of Panel meetings and usability testing sessions will be deleted once analyzed and/or transcribed and transcription texts will be deidentified. No names, addresses, or other identifying information will be recorded in this data file. For reporting purposes, your answers will be combined with the answers from the other respondents and will appear only as aggregated information. Participants will have the opportunity to engage in dissemination activities at their discretion.

The servers where the data will be stored are controlled and monitored by the NUIT Research Data Center. The data center has controlled access, and the server is on secure data networks behind managed firewalls. The server is also monitored for service interruptions. Access to the data is only allowed from NU networks and is encrypted so that only the staff on our team have access to the data.

Study records that can identify you are kept confidential through use of a participant identification number. All information will be kept on a password protected computer only accessible by the research team.

Certificate of Confidentiality:

This research is covered by a Certificate of Confidentiality from the National Institutes of Health. This means that the researchers cannot release or use information, documents, or samples that may identify you in any action or suit unless you say it is okay. They also cannot provide them as evidence unless you have agreed. This protection includes federal, state, or local civil, criminal, administrative, legislative, or other proceedings. An example would be a court subpoena.

Identifiable information that could still be disclosed beyond the research team: The Certificate does not stop reporting that federal, state or local laws require. Some examples are laws that require reporting of child or elder abuse, some communicable diseases, and threats to harm yourself or others. The Certificate cannot be used to stop a sponsoring United States federal or state government agency from checking records or evaluating programs. The Certificate does not stop disclosures required by the federal Food and Drug Administration (FDA). The Certificate also does not prevent your information from being used for other research if allowed by federal regulations.

Researchers may release information about you when you say it is okay. For example, you may give them permission to release information to insurers, medical providers or any other persons not connected with the research. The Certificate of Confidentiality does not stop you from willingly releasing information about your involvement in this research. It also does not prevent you from having access to your own information.

Who will have access to the information collected during this research study?

Efforts will be made to limit the use and disclosure of your personal information, including research study records, to people who have a need to review this information. We cannot promise complete secrecy.

There are reasons why information about you may be used or seen by other people beyond the research team during or after this study. Examples include:

- University officials, government officials, study funders, auditors, and the Institutional Review Board may need access to the study information to make sure the study is done in a safe and appropriate manner.
- Collaborating researchers at other institutions who are involved with this study.
- The research team may give information to appropriate authorities for reasons of health and safety – for example, if you indicate that you plan to harm yourself or others, or for public health reasons.

How might the information collected in this study be shared in the future?

We will keep the information we collect about you during this research study for study recordkeeping and potential use in future research projects. De-identified data from this study may be shared with the research community, with journals in which study results are published, and with databases and data repositories used for research. We will remove any personal information that could directly identify you before the study data are shared. Despite these measures, we cannot guarantee anonymity of your personal data. The results of this study could be shared in articles and presentations, but will not include any information that identifies you unless you give permission for use of information that identifies you in articles and presentations, only as applicable.

Will I be paid or given anything for taking part in this study?

The study will provide either (a) $3,000 of your salary at your CHC plus 18% fringe for a total of $3,540, payable directly to your CHC (this is a rate of 1.56% FTE at the 2019 NIH salary cap rate of $192,300), OR (b) $3,000 in stipend payable to you (will require completion of a Form 1099 and registration with Northwestern University as a vendor). Transportation and parking costs for attendance at in-person meetings will also be covered.The study number for this project is: STU00210809.

Who can I talk to?

If you have questions, concerns, or complaints, you can contact Justin D. Smith, at (312) 503-4041 and jd.smith@northwestern.edu. This research has been reviewed and approved by an Institutional Review Board ("IRB") – an IRB is a committee that protects the rights of people who participate in research studies. You may contact the IRB by phone at (312) 503-9338 or by email at irb@northwestern.edu if:

- Your questions, concerns, or complaints are not being answered by the research team.
- You cannot reach the research team.
- You want to talk to someone besides the research team.
- You have questions about your rights as a research participant.
- You want to get information or provide input about this research.

Your signature documents your permission to take part in this research.

____ ________________
Signature of participant Date

____
Printed name of participant

____ ________________
Signature of person obtaining consent Date

____
Printed name of person obtaining consent

Title of Research Study: Implementation Preparation of a Population Panel Management Intervention in Safety-Net Clinics for Childhood Hypertension

Principal Investigator: Justin D. Smith, PhD, Associate Professor in the Department of Psychiatry and Behavioral Sciences

Supported By: This research is supported by Northwestern University and the National Heart, Lung, and Blood Institute.

Key Information about this research study:

The following is a short summary of this study to help you decide whether to be a part of this study. Information that is more detailed is explained later on in this form.

- This study is part of a series of studies aimed at adapting Health Information Technology (HIT) tools to help diagnose and manage abnormal blood pressure of youth ages 3 to 17 years.
- The purpose of this study is to evaluate the usability of a population health tool and related HIT (e.g., clinical decision supports) currently under development over two sessions.
- You will be asked to complete two separate sessions with an interview with a member of the research staff, complete tasks on the tool, and answer questionnaires about your experience using the tool.
- We expect that the two sessions will take about 50 mintues each.
- Your participation does not involve any significant risks. There is a potential risk of discomfort, as some of the questions are about personal professional practices and views.
- You are not likely to have any direct benefit from being in this research study. One potential benefit is that the knowledge gained from this study may help inform the design of HIT tool for diagnosing and managing blood pressure.

Why am I being asked to take part in this research study?

We are asking you to take part in this research study because you are a primary care provider who provides care to children and youth.

How many people will be in this study?

We expect about 10 people will be in this research study.

What should I know about participating in a research study?

- Someone will explain the research study to you.
- Whether or not you take part is up to you.
- You can choose not to take part.
- You can agree to take part and later change your mind.
- Your decision will not be held against you.
- You can ask all the questions you want before you decide.

What happens if I say, "Yes, I want to be in this research"?

If you are eligible to participate in the study, you will participate in two sessions scheduled on two separate days. Each session will include a semi-structured interview with a member of the research staff in which you will review the population health tool and related HIT (e.g., clinical decision supports) currently under development and complete tasks using the tool. We will ask to video record the session as a requirement of your participation so that the study team may later transcribe the

session, which aids with data analysis. After completing tasks, you will be asked about your perceptions of an experience with the tool.

You will be asked to complete this interview in person at a local community health center. The interviews will be video and audio recorded. These recordings will allow researchers to go back and review the sessions and information to inform the development of the technology.

Is there any way being in this study could be bad for me?

Your participation does not involve any significant risks. There is a potential risk of discomfort, as some of the questions are about personal professional practices and views. You can skip any activities of the study you do not wish to participate in, or withdrawal from the study at any point.

What happens if I do not want to be in this research, or I change my mind later?

Participation in research is voluntary. You can decide to participate or not to participate. If you do not want to be in this study or withdraw from the study at any point, your decision will not affect your relationship with Northwestern University. You can leave the research at any time and it will not be held against you. If you decide to withdraw from this study, the researchers will ask you if information already collected from you can be used.

How will the researchers protect my information?

If you decide to participate, all of your responses will be kept confidential. All identifying information will be removed from the data collected, which will include qualitative data and survey responses. A de-identified data file will be prepared from the survey data of all respondents and used for analysis and may be shared with the study sponsor. Audio and video recordings of usability testing sessions will be deleted once analyzed and/or transcribed and transcription texts will be deidentified. No names, addresses, or other identifying information will be recorded in this data file. For reporting purposes, your answers will be combined with the answers from the other respondents and will appear only as aggregated information. Participants will have the opportunity to engage in dissemination activities at their discretion.

The servers where the data will be stored are controlled and monitored by the NUIT Research Data Center. The data center has controlled access, and the server is on secure data networks behind managed firewalls. The server is also monitored for service interruptions. Access to the data is only allowed from NU networks and is encrypted so that only the staff on our team have access to the data. Study records that can identify you are kept confidential through use of a participant identification number. All information will be kept on a password protected computer only accessible by the research team.

Certificate of Confidentiality:

This research is covered by a Certificate of Confidentiality from the National Institutes of Health. This means that the researchers cannot release or use information, documents, or samples that may identify you in any action or suit unless you say it is okay. They also cannot provide them as evidence unless you have agreed. This protection includes federal, state, or local civil, criminal, administrative, legislative, or other proceedings. An example would be a court subpoena.

Identifiable information that could still be disclosed beyond the research team: The Certificate does not stop reporting that federal, state or local laws require. Some examples are laws that require reporting of child or elder abuse, some communicable diseases, and threats to harm yourself or others. The Certificate cannot be used to stop a sponsoring United States federal or state government agency from

checking records or evaluating programs. The Certificate does not stop disclosures required by the federal Food and Drug Administration (FDA). The Certificate also does not prevent your information from being used for other research if allowed by federal regulations.

Researchers may release information about you when you say it is okay. For example, you may give them permission to release information to insurers, medical providers or any other persons not connected with the research. The Certificate of Confidentiality does not stop you from willingly releasing information about your involvement in this research. It also does not prevent you from having access to your own information.

Who will have access to the information collected during this research study?

Efforts will be made to limit the use and disclosure of your personal information, including research study records, to people who have a need to review this information. We cannot promise complete secrecy.

There are reasons why information about you may be used or seen by other people beyond the research team during or after this study. Examples include:

- University officials, government officials, study funders, auditors, and the Institutional Review Board may need access to the study information to make sure the study is done in a safe and appropriate manner.
- Collaborating researchers at other institutions who are involved with this study.
- The research team may give information to appropriate authorities for reasons of health and safety – for example, if you indicate that you plan to harm yourself or others, or for public health reasons.

How might the information collected in this study be shared in the future?

We will keep the information we collect about you during this research study for study recordkeeping and potential use in future research projects. De-identified data from this study may be shared with the research community, with journals in which study results are published, and with databases and data repositories used for research. We will remove any personal information that could directly identify you before the study data are shared. Despite these measures, we cannot guarantee anonymity of your personal data. The results of this study could be shared in articles and presentations, but will not include any information that identifies you unless you give permission for use of information that identifies you in articles and presentations, only as applicable.

Will I be paid or given anything for taking part in this study?

If you agree to take part in this research study, we will pay you $75 for each session ($150 total). You will be paid at the end of each session and you will be paid with a visa gift card. Transportation and parking costs for attendance at in-person sessions will also be covered. The study number for this project is: STU00210809.

Who can I talk to?

If you have questions, concerns, or complaints, you can contact Justin D. Smith, at (312) 503-4041 and jd.smith@northwestern.edu. This research has been reviewed and approved by an Institutional Review Board ("IRB") – an IRB is a committee that protects the rights of people who participate in research studies. You may contact the IRB by phone at (312) 503-9338 or by email at irb@northwestern.edu if:

- Your questions, concerns, or complaints are not being answered by the research team.
- You cannot reach the research team.
- You want to talk to someone besides the research team.
- You have questions about your rights as a research participant.
- You want to get information or provide input about this research.

Your signature documents your permission to take part in this research.

____ ____________________
Signature of participant Date

____
Printed name of participant

____ ____________________
Signature of person obtaining consent Date

____
Printed name of person obtaining consent

Title of Research Study: Implementation Preparation of a Population Panel Management Intervention in Safety-Net Clinics for Childhood Hypertension

Principal Investigator: Justin D. Smith, PhD, Associate Professor in the Department of Psychiatry and Behavioral Sciences

Supported By: This research is supported by Northwestern University and the National Heart, Lung, and Blood Institute.

Key Information about this research study:

The following is a short summary of this study to help you decide whether to be a part of this study. Information that is more detailed is explained later on in this form.

- The purpose of this study is to learn more about how primary care providers view and screen for chronic health conditions, such as hypertension/uncontrolled blood pressure, and the social-wellbeing of children and whether health information technology tools could be modified or designed to better support practicing primary care providers.
- You will be asked to complete an online survey.
- We expect that you will be in this research study for 15-20min.
- Your participation does not involve any significant risks. There is a potential risk of discomfort, as some of the questions are about personal professional practices and views.
- There are no direct benefits for participation. One potential benefit is that the knowledge gained from this study may contribute to a better understanding of child and adolescent chronic health conditions and social-emotional wellbeing and may help inform the design of technology tools to better support practicing primary care providers.
- We expect about 300 medical health care providers will be in this research study.

Why am I being asked to take part in this research study?

We are asking you to take part in this research study because you are a primary care provider who provides care to children and youth.

How many people will be in this study?

We expect about 300 people will be in this research study.

What should I know about participating in a research study?

- Whether or not you take part is up to you.
- You can choose not to take part.
- You can agree to take part and later change your mind.
- Your decision will not be held against you.
- You do not have to answer any question you do not want to answer.

What happens if I say, "Yes, I want to be in this research"?

If you agree to participate, you will be asked to complete an online survey regarding your perspective and measurement/screening of chronic health conditions, such as hypertension/uncontrolled blood pressure, and social-wellbeing of children; and whether the design of health information technology tools would better meet your needs and support you in primary care.

Is there any way being in this study could be bad for me?

Your participation does not involve any significant risks. There is a potential risk of discomfort, as some of the questions are about personal professional practices and views. You can skip any question you do not wish to answer, or exit the survey at any point.

What happens if I do not want to be in this research, or I change my mind later?

Participation in research is voluntary. You can decide to participate or not to participate.If you do not want to be in this study or withdraw from the study at any point, your decision will not affect your relationship with Northwestern University. You can leave the research at any time and it will not be held against you. If you decide to withdraw from this study, the answers already collected up to that point will be used.

How will the researchers protect my information?

This survey is being hosted by REDCap (Research Electronic Data Capture) and involves a secure connection. The servers where the data is stored are controlled and monitored by the NUIT Research Data Center. The data center has controlled access, and the server is on secure data networks behind managed firewalls. The server is also monitored for service interruptions. Access to the data is only allowed from NU networks and is encrypted so that only the staff on our team have access to the data.

Study records that can identify you are kept confidential through use of a participant identification number. Your email address will be stored separately from your survey data, and is only being collected for potential payment purposes. All information will be kept on a password protected computer only accessible by the research team. The results of the research study may be published, but your name will not be used.

Certificate of Confidentiality:

This research is covered by a Certificate of Confidentiality from the National Institutes of Health. This means that the researchers cannot release or use information, documents, or samples that may identify you in any action or suit unless you say it is okay. They also cannot provide them as evidence unless you have agreed. This protection includes federal, state, or local civil, criminal, administrative, legislative, or other proceedings. An example would be a court subpoena.

Identifiable information that could still be disclosed beyond the research team: The Certificate does not stop reporting that federal, state or local laws require. Some examples are laws that require reporting of child or elder abuse, some communicable diseases, and threats to harm yourself or others. The Certificate cannot be used to stop a sponsoring United States federal or state government agency from checking records or evaluating programs. The Certificate does not stop disclosures required by the federal Food and Drug Administration (FDA). The Certificate also does not prevent your information from being used for other research if allowed by federal regulations.

Researchers may release information about you when you say it is okay. For example, you may give them permission to release information to insurers, medical providers or any other persons not connected with the research. The Certificate of Confidentiality does not stop you from willingly releasing information about your involvement in this research. It also does not prevent you from having access to your own information.

Who will have access to the information collected during this research study?

Efforts will be made to limit the use and disclosure of your personal information, including research study records, to people who have a need to review this information. We cannot promise complete secrecy.

There are reasons why information about you may be used or seen by other people beyond the research team during or after this study. Examples include:

- University officials, government officials, study funders, auditors, and the Institutional Review Board may need access to the study information to make sure the study is done in a safe and appropriate manner.
- Collaborating researchers at other institutions who are involved with this study.
- The research team may give information to appropriate authorities for reasons of health and safety – for example, if you indicate that you plan to harm yourself or others, or for public health reasons.

How might the information collected in this study be shared in the future?

We will keep the information we collect about you during this research study for study recordkeeping and potential use in future research projects. De-identified data from this study may be shared with the research community, with journals in which study results are published, and with databases and data repositories used for research. We will remove any personal information that could directly identify you before the study data are shared. Despite these measures, we cannot guarantee anonymity of your personal data. The results of this study could be shared in articles and presentations, but will not include any information that identifies you unless you give permission for use of information that identifies you in articles and presentations, only as applicable.

Will I be paid or given anything for taking part in this study?

If you agree to take part in this research study, we will provide you with up to $15 in visa gift cards sent to the email address you provide at the end of the survey. You will receive $10 for completing the first part of the survey, and then $5 for completing the second part. The study number for this project is: STU00210809.

Who can I talk to?

If you have questions, concerns, or complaints, you can contact the Principal Investigator, Justin D. Smith, at (312) 503-4041 and jd.smith@northwestern.edu. This research has been reviewed and approved by an Institutional Review Board ("IRB") – an IRB is a committee that protects the rights of people who participate in research studies. You may contact the IRB by phone at (312) 503-9338 or by email at irb@northwestern.edu if:

- Your questions, concerns, or complaints are not being answered by the research team.
- You cannot reach the research team.
- You want to talk to someone besides the research team.
- You have questions about your rights as a research participant.
- You want to get information or provide input about this research.

Online Consent

If you want a copy of this consent for your records, you can print it from the screen. If you wish to participate, please click the "I Agree" button and you will be taken to the survey. If you do not wish to participate in this study, please select "I Disagree" or select X in the corner of your browser.

Title of Research Study: Implementation Preparation of a Population Panel Management Intervention in Safety-Net Clinics for Childhood Hypertension

Principal Investigator: Justin D. Smith, PhD, Associate Professor in the Department of Psychiatry and Behavioral Sciences

Supported By: This research is supported by Northwestern University and the National Heart, Lung, and Blood Institute.

Key Information about this research study:

The following is a short summary of this study to help you decide whether you want to participate as well as whether you want to permit your child to be a part of this study.

- The purpose of this study is to learn from parents and youth who receive care from a pediatric healthcare provider about their thoughts and perspective around heart health for youth.
- You and your child will be asked to participate in an interview.
- We expect that you and your child will be in the research study for 45-60 minutes.
- There are no foreseeable risks for you and your child to participate in this study; although some parents and youth may feel uncomfortable with some of the topics around heart health, such as high blood pressure.
- There are no direct benefits for participation. One potential benefit is that the knowledge gained from this study may contribute to a better understanding of child and adolescent heart health and may help inform the design of digital tools to better evaluate heart health in children and adolescents.
- We expect about 20 parents and youth (aged 10-17 years) will be in this research study.
- You and your child can ask all the questions you want before you both decide whether or not to participate.

If you say that "Yes, you want you and your child to be in this research," here is what each of you will be asked to do:

If you provide permission for you and your child to join this study, you and your child will be asked to participate in a one-time, in-person interview. This session will last between 45 and 60 minutes. We will schedule the session at the Chicago campus of Northwestern University, fitting in with your family's schedule. We will ask to audio record the session as a requirement of you and your child's participation so that the study team may later transcribe the session, which aids with data analysis. You and your child will meet with an interviewer at a community location or at Northwestern University. The researcher will notify you and your child when the audio recorder has been turned on, and when it is turned off.

Is there any way being in this study could be bad for me or my child?

There is nothing bad that will happen to you and your child, although you or your child may feel uncomfortable with some of the topics we ask about related to heart health, such as high blood pressure. You and your child can skip any questions you do not want to answer and can stop at any time.

If you say that you do not want you or your child to be in this research:

Participation in research is completely voluntary. You can decide if you do not want you or your child to participate in this research and it will not be held against you or your child in any way.

You can say "Yes," but change your mind later:

You or your child can stop and leave the research at any time and it will not be held against either of you. We can end the interview at any time. Just let me know if you want to stop. If this happens, I will ask you if any data collected from you or your child up until that point may be used in the research.

This is what will happen to the information collected for this research:

De-identified data from this study may be shared with the research community at large to advance science and health. We will remove or code any personal information that could identify you and your child before files are shared with other researchers to ensure that, by current scientific standards and known methods, no one will be able to identify you and your child from the information we share. We cannot guarantee anonymity of you and your child's personal data despite these measures; however, I will work hard to keep you and your child's name and other information private. Additionally, efforts will be made to limit the use and disclosure of you and your child's personal information, including research study records, to people who have a need to review this information, such as the Institutional Review Board.

I will not ask you or your child about abuse, but if you or your child tells me plans to hurt yourself or someone else, or that someone is hurting or neglecting you, I have to do whatever is needed to keep everyone safe, which may require notifying the authorities and/or others. We are legally obligated to report child abuse or neglect to state authorities.

I will retain audio files and transcriptions of the feedback sessions for no longer than 10 years after the end of the study. I will also retain this parent/guardian permission with child assent form with participant names. All types of data will be secured using passwords (for computer-based data) or secured in a locked cabinet (for paper data), and available only to researchers approved to work on this project. The information gathered from the interview will be kept separate from information which identifies you and your child.

Certificate of Confidentiality:

This research is covered by a Certificate of Confidentiality from the National Institutes of Health. This means that the researchers cannot release or use information, documents, or samples that may identify you in any action or suit unless you say it is okay. They also cannot provide them as evidence unless you have agreed. This protection includes federal, state, or local civil, criminal, administrative, legislative, or other proceedings. An example would be a court subpoena.

Identifiable information that could still be disclosed beyond the research team: The Certificate does not stop reporting that federal, state or local laws require. Some examples are laws that require reporting of child or elder abuse, some communicable diseases, and threats to harm yourself or others. The Certificate cannot be used to stop a sponsoring United States federal or state government agency from checking records or evaluating programs. The Certificate does not stop disclosures required by the federal Food and Drug Administration (FDA). The Certificate also does not prevent your information from being used for other research if allowed by federal regulations.

Researchers may release information about you when you say it is okay. For example, you may give them permission to release information to insurers, medical providers or any other persons not connected with the research. The Certificate of Confidentiality does not stop you from willingly releasing information about your involvement in this research. It also does not prevent you from having access to your own information.

Here is some other information that is useful for you and your child to know:

If you agree for you and your child to take part in this study, we will provide compensation of two $20 Visa Gift Cards, one for you and one for your child ($40 total) for your efforts and time at the end of the interview. You and your child will still receive this compensation even if participation is ended early.

Here is who you and your child can talk to:

If you have questions, concerns, or complaints, you can talk to the Principal Investigator, Justin D. Smith, at (312) 503-4041 and jd.smith@northwestern.edu.This research has been reviewed and approved by an Institutional Review Board. You may talk to them at (312) 503-9338 or irb@northwestern.edu if:

- Your questions, concerns, or complaints are not being answered by the research team.
- You cannot reach the research team.
- You want to talk to someone besides the research team.
- You have questions about your rights as a research participant.
- You want to get information or provide input about this research.

Your signature documents your consent to participate and your permission for the named child to take part in this research.

____ ______________

Signature of child Date

____

Printed name of child

____ ______________

Printed name of parent [ ] or individual legally authorized [ ] Date
to consent for the child to participate

____ ______________

Signature of parent [ ] or individual legally authorized [ ] Date
to consent for the child to participate

My signature below documents that the information in the permission document and any other written and verbal information was accurately explained to, and apparently understood by, the parent and child, and that permission was freely given by the parent for their own participation and for their child's participation in this study, and the child's assent was freely given for their own participation in this research study.

____ ______________

Signature of research personnel obtaining parent permission Date

____

Printed name of research personnel obtaining parent permission

Title of Research Study: Implementation Preparation of a Population Panel Management Intervention in Safety-Net Clinics for Childhood Hypertension

Principal Investigator: Justin D. Smith, PhD, Associate Professor in the Department of Psychiatry and Behavioral Sciences

Supported By: This research is supported by Northwestern University and the National Heart, Lung, and Blood Institute.

Key Information about this research study:

The following is a short summary of this study to help you decide whether you want to participate as well as whether you want to permit your child to be a part of this study.

- The purpose of this study is to learn from parents and youth who receive care from a pediatric healthcare provider about their thoughts and perspective around heart health for youth.
- You and your child will be asked to participate in an interview.
- We expect that you and your child will be in the research study for 45-60 minutes.
- There are no foreseeable risks for you and your child to participate in this study; although some parents and youth may feel uncomfortable with some of the topics around heart health, such as high blood pressure.
- There are no direct benefits for participation. One potential benefit is that the knowledge gained from this study may contribute to a better understanding of child and adolescent heart health and may help inform the design of digital tools to better evaluate heart health in children and adolescents.
- We expect about 20 parents and youth (aged 10-17 years) will be in this research study.
- You and your child can ask all the questions you want before you both decide whether or not to participate.

If you say that "Yes, you want you and your child to be in this research," here is what each of you will be asked to do:

If you provide permission for you and your child to join this study, you and your child will be asked to participate in a one-time, in-person interview. This session will last between 45 and 60 minutes. We will schedule the session at the Chicago campus of Northwestern University, fitting in with your family's schedule. We will ask to audio record the session as a requirement of you and your child's participation so that the study team may later transcribe the session, which aids with data analysis. You and your child will meet with an interviewer at a community location or at Northwestern University. The researcher will notify you and your child when the audio recorder has been turned on, and when it is turned off.

Is there any way being in this study could be bad for me or my child?

There is nothing bad that will happen to you and your child, although you or your child may feel uncomfortable with some of the topics we ask about related to heart health, such as high blood pressure. You and your child can skip any questions you do not want to answer and can stop at any time.

If you say that you do not want you or your child to be in this research:

Participation in research is completely voluntary. You can decide if you do not want you or your child to participate in this research and it will not be held against you or your child in any way.

You can say "Yes," but change your mind later:

You or your child can stop and leave the research at any time and it will not be held against either of you. We can end the interview at any time. Just let me know if you want to stop. If this happens, I will ask you if any data collected from you or your child up until that point may be used in the research.

This is what will happen to the information collected for this research:

De-identified data from this study may be shared with the research community at large to advance science and health. We will remove or code any personal information that could identify you and your child before files are shared with other researchers to ensure that, by current scientific standards and known methods, no one will be able to identify you and your child from the information we share. We cannot guarantee anonymity of you and your child's personal data despite these measures; however, I will work hard to keep you and your child's name and other information private. Additionally, efforts will be made to limit the use and disclosure of you and your child's personal information, including research study records, to people who have a need to review this information, such as the Institutional Review Board.

I will not ask you or your child about abuse, but if you or your child tells me plans to hurt yourself or someone else, or that someone is hurting or neglecting you, I have to do whatever is needed to keep everyone safe, which may require notifying the authorities and/or others. We are legally obligated to report child abuse or neglect to state authorities.

I will retain audio files and transcriptions of the feedback sessions for no longer than 10 years after the end of the study. I will also retain this parent/guardian permission with child assent form with participant names. All types of data will be secured using passwords (for computer-based data) or secured in a locked cabinet (for paper data), and available only to researchers approved to work on this project. The information gathered from the interview will be kept separate from information which identifies you and your child.

Certificate of Confidentiality:

This research is covered by a Certificate of Confidentiality from the National Institutes of Health. This means that the researchers cannot release or use information, documents, or samples that may identify you in any action or suit unless you say it is okay. They also cannot provide them as evidence unless you have agreed. This protection includes federal, state, or local civil, criminal, administrative, legislative, or other proceedings. An example would be a court subpoena.

Identifiable information that could still be disclosed beyond the research team: The Certificate does not stop reporting that federal, state or local laws require. Some examples are laws that require reporting of child or elder abuse, some communicable diseases, and threats to harm yourself or others. The Certificate cannot be used to stop a sponsoring United States federal or state government agency from checking records or evaluating programs. The Certificate does not stop disclosures required by the federal Food and Drug Administration (FDA). The Certificate also does not prevent your information from being used for other research if allowed by federal regulations.

Researchers may release information about you when you say it is okay. For example, you may give them permission to release information to insurers, medical providers or any other persons not connected with the research. The Certificate of Confidentiality does not stop you from willingly releasing information about your involvement in this research. It also does not prevent you from having access to your own information.

Here is some other information that is useful for you and your child to know:

If you agree for you and your child to take part in this research study, we will provide compensation of two $20 Visa Gift Cards, one for you and one for your child ($40 total) for your efforts and time at the end of the interview. You and your child will still receive this compensation even if participation is ended early.

Here is who you and your child can talk to:

If you have questions, concerns, or complaints, you can talk to the Principal Investigator, Justin D. Smith, at (312) 503-4041 and jd.smith@northwestern.edu.This research has been reviewed and approved by an Institutional Review Board ("IRB"). You may talk to them at (312) 503-9338 or irb@northwestern.edu if:

- Your questions, concerns, or complaints are not being answered by the research team.
- You cannot reach the research team.
- You want to talk to someone besides the research team.
- You have questions about your rights as a research participant.
- You want to get information or provide input about this research.

Your signature documents your permission for the named child to take part in this research.

____
Printed name of child

____ _______________
Printed name of parent [ ] or individual legally authorized [ ] to consent for the child to participate — Date

____ _______________
Signature of parent [ ] or individual legally authorized [ ] to consent for the child to participate — Date

My signature below documents that the information in the permission document and any other written and verbal information was accurately explained to, and apparently understood by, the parent and child, and that permission was freely given by the parent for their own participation and for their child's participation in this study, and the child's assent was freely given for their own participation in this research study.

____ _______________
Signature of research personnel obtaining parent permission — Date

____
Printed name of research personnel obtaining parent permission

Name______________________ ID Number__________________

CHILD ASSENT
Children and Adolescent Heart Health Study
Northwestern University

Introduction

Your Mom/Dad said it's okay for you to take part in this study about your health. You are invited to be part of a research study on heart health and blood pressure. Your heart is a muscle that sends blood around your body. One way to check how hard your heart is pumping to move blood through your body is through checking your blood pressure. You might have had your blood pressure checked when you went to the doctor for a checkup. To check your blood pressure, a nurse might put a band around part of your arm and pump air into the band, blowing it up like a balloon. Your arm might have felt a little squished, but don't worry – that's how a nurse checks your blood pressure. Researcher Dr. Justin Smith at Northwestern University is doing this study with funding from the National Heart, Lung, and Blood Institute. The goal of the study is to learn from you about your checkups with your doctor. We also want to learn your ideas on heart health and blood pressure. This assent tells you about the study so you can decide if you want to be part of it. If you say "yes," your agreement will be recorded on this form.

Description of the Study

This session should take about between 45 and 60 minutes and as a thank you for helping us, you will receive a gift of a $20 Visa Gift Card.

During the session, you will be asked questions about your checkups with the doctor, about your heart, and checking your blood pressure. You can say that you don't want to answer a question and you can skip any questions that you don't want to answer. It's also ok if you want to end the interview and stop talking to the study team member at any time.

Your answers will not be shared with other people. We have something called a Certificate of Confidentiality. This means that we cannot be forced to tell anyone what you share with us, even if a court asks for it. There are two times where we would share what you tell us with someone. Those are:

1) If during the interview you share with us that you are feeling really sad and we think you might need some help to deal with your feelings, then we will talk with your parent and other professionals about getting you the help you need.
2) If we learn that you or another person is being seriously hurt or threatened or has been seriously hurt in the past, then we would take steps to protect that person.
   Check this box if you have read the above assent or it has been read to you, are over 9 years old and agree to participate in the study we described. Checking this box means that you acknowledge that we explained the nature and demands of this study and the possible negative effects and benefits. You accept these risks and understand that you can stop being part of the study at any time without penalty. We will give you a copy of this form.

____ __________________
Signature of Child Date

____
Printed name of Child

____ __________________
Printed name of person obtaining assent Date

____
Signature of person obtaining assent
